# Supplementary material for: The Product of the Fission Yeast fhl1 Gene Binds to the HomolE Box and Activates In Vitro Transcription of Ribosomal Protein Genes
Source: Int J Mol Sci. 2023 May 30;24(11):9472. doi: 10.3390/ijms24119472 (PMC10253590; doi:10.3390/ijms24119472)
Supplement: Supplementary file 1 [file ijms-24-09472-s001.zip › ijms-2405036-Figure S1.pdf]

```
Smittium culisis               MDDKNAATVLAERFSSGNYDYSDSNTPNLLPRGTPSRRSKRKSKADTTLTNPKRHVASKL 60
Pneumocystis murina            ------------------------------------------------------------ 0
Schizosaccharomyces pombe      ------------------------------------------------------------ 0
Schizosaccharomyces octosporus ------------------------------------------------------------ 0

Smittium culisis               SSTGNTDCFPKFGLLPPKDQQMDSFSHSKSNNIIPTSFNNVFSYHSIDNSGRPNNPYLNT 120
Pneumocystis murina            ------------------------------------------------------------ 0
Schizosaccharomyces pombe      ------------------------------------------------------------ 0
Schizosaccharomyces octosporus ------------------------------------------------------------ 0

Smittium culisis               TNQIINDMKNHTSLHDTIYDHNTQYNPNKDNNTSFDIQNQRFSFNDPHQKLSMPENFPEK 180
Pneumocystis murina            ------------------------------------------------------------ 0
Schizosaccharomyces pombe      ------------------------------------------------------------ 0
Schizosaccharomyces octosporus ------------------------------------------------------------ 0

Smittium culisis               ILCQDFEHYYAN--DYYPYENPINTFQEVEDQPVQAFAKLEGPNFNYYIKAISVTLGRQA 238
Pneumocystis murina            MTINTFEMQSKNSLDIMIKNEDTGIIEPQPSRNVQAYAKLEFDAFSFYIQTLQVIIGRKV 60
Schizosaccharomyces pombe      ---------------MPVAEIKNATQQPSSTNRVQAYAKLEFEKFSFFVQTLQVTMGRKA 45
Schizosaccharomyces octosporus ---------------MPDAVRQDAAEQKSGSNRVQAYAKLEFEKFSFFVQTLQVTMGRKA 45
                                                         :    . ***:****   *.::::::.* :**:.

Smittium culisis               TSQELADIILGENKALSRKHARIFYNFMSQGFELQVFGKNGCFVDGLFIQKGATVPLNHR 298
Pneumocystis murina            NKLDQVDVHIGSTKAISRQHAKLFYDFTSQRFEIFVMGKNGAFINEEFVECGQTIPLYDK 120
Schizosaccharomyces pombe      SNSSDCDVHLGDTKAISRQHAKIFYSFPNQRFEISVMGKNGAFVDGEFVERGKSVPLRSG 105
Schizosaccharomyces octosporus SNFSDCDVHLGDTKAISRQHAKIVYNFPNQRFEFSVIGKNGAFVDGEFVERGSTVPLHSG 105
                               .. .  *: :*..**:**:**::.*.* .* **: *:****.*::  *:: * ::**

Smittium culisis               TIIMMGDSCFTFLLPKNSIPAQHETASTTGT--LS---AEIAYEHQEIDH---------- 343
Pneumocystis murina            TKIQIGKVLFTFLLPKSTEKENTENRDIREN--QRIEKS-------EFSQ---------- 161
Schizosaccharomyces pombe      TRVQIGQISFSFLLPEGSEEDGHLKETGIT--PLSLQQGKIAYSD-EFGGKPTGSFHTVT 162
Schizosaccharomyces octosporus TRVQIGQISFSFLLPEGMQKQKAEKHDADTADPQKMNTT---VTP-VIEQKPP------- 154
                               * : :*.  *:****:.       .                      :

Smittium culisis               --QNNNLRFKN---NKGL-----A-------------NYQYQDNELPFQDDDNTFYNEND 380
Pneumocystis murina            ---DISLLSNSVSTERS--------------------LVTYPNINL-----ELSLIKTDE 193
Schizosaccharomyces pombe      SNQEKDLLFSHIKHESDL-PLGLSP--ADTNISNATSIIEHPDAAN-----AHTLASLNQ 214
Schizosaccharomyces octosporus IEELQSSFYTSVKKESELNPTHLSPPPLMTSEATSANIFEHPDAIP-----PRPLESFPY 209
                                    .   .    :                         : :           : .

Smittium culisis               PNK--RMSINSESLVRDTSTG------SETPTVLDSNMQSTP-----ISQSYLNGGPGT- 426
Pneumocystis murina            PD------ILPISSRI----------------LNNE-TLSAV--NSTFHDESLL------ 222
Schizosaccharomyces pombe      PPK--HLTVSPSSIQRLSPQPYVRPTSDERPIETDS-SVSAP--KVANHDEELKQGKSTS 269
Schizosaccharomyces octosporus PSEYKPQTVSPQSIQRQTSSDFSSAPSANPETVRNF-SETPPANTIPPKQIPVDQESHRI 268
                               *       :   *                     :    :         :  :

Smittium culisis               -DMVRILPSTAKPQLPNNGKVFTKPTFSYASLIAQAINSTEEKKITLNGIYTYIMSNFPY 485
Pneumocystis murina            -----SHA--E--SFNVSMKEHSKPNLSYASLIAQAILSSPSKKMTLSDIYEWITDTYKY 273
Schizosaccharomyces pombe      PSDTVLHPDLN--GSPDTGDATQKPNLSYANLIARTLIANPNKKMTLGDICEWIANNWSY 327
Schizosaccharomyces octosporus STSIPLHPGLESQPPPNLGDIYQKPNLSYANLIARTLISNSNKKMTLGDICEWISHTWPY 328
                                                  .   **.:***.***::: :. .**:**..*  :*  .: *

Smittium culisis               YREAQNGWQNSIRHNLSLNKAFEKVQRESNQPGKGSYWKICDNFVNQFDNGVYKRMRRTS 545
Pneumocystis murina            YKYAQNGWQNSIRHSLSLNKAFKKVSRRDDEPGKGSFWMINSEYQCQFEDGIYKRNRKSI 333
Schizosaccharomyces pombe      YRHQPPAWHNSIRHNLSLNKAFIRIPRRQNEPGKGSFWMLDPSYIDQFEGNFFRRTKKPT 387
Schizosaccharomyces octosporus YQYQPPAWHNSIRHNLSLNKAFIRIPRRQNESGKGSFWILDPSYIDQFEGNMFRKTKKPT 388
                               *:    .*:*****.******* :: *..:: ****:* :  .:  **:...::: ::

Smittium culisis               KKKSTDSTISAK--------SSSKTLTGSSQKKNKNDYHNSNIIDTPSKPPTGT------ 591
Pneumocystis murina            ISSYQHLT--------TPSIMADG------------------------------------ 349
Schizosaccharomyces pombe      PSATPAAHPDTARENELAAIQTKGISAGKTEQLNP--------QKETSRSKTHTSRGENV 439
Schizosaccharomyces octosporus PTATGATAFEHENIDPLRSTMSAGGKMGSSK---T--------SDTSQRAPTVNGGAAAS 437
                                .                   :

Smittium culisis               -KNAQSLLTPPISQELLDN-----QPDL-------EDSVSISSSSVKSFYSNDPNNSFDD 638
Pneumocystis murina            ---ASRSTADSIPIAIMQDGQLALNPEYFNTVNDSNDSNKIQIVQAIVFLQRYIITQLGP 406
Schizosaccharomyces pombe      EDRPQSLLQNGIQPIIMRDGKLALNPEFFKNANGEQQAPNEQAVQAISLLQQHINKQLGP 499
Schizosaccharomyces octosporus KEEKSSLLQSGIQPIIMQNGKLALNPEFFRNSNGEQQAPNEQAVHAISLLQDHINRQLGP 497
                                   .      *   :: :     :*:        ::: . .   .  : .     .:.

Smittium culisis               TKKKYTIQPNIPDNSTMN-SSLIQP----------------------------------- 662
Pneumocystis murina            ------HAK-IPQNAAAIANALIVALDQQLQKHQNHKNFISNIFKNLTFLLPNTNEKMHT 459
Schizosaccharomyces pombe      ------AAANNPEQATAIANALAVALAQKLQKQQTQMQGPQQV-QQQ-------AKRRK- 544
Schizosaccharomyces octosporus ------AAVNNPAQATAIANALAVALAKKLQKQPVPAAPVQQDTGQQ-------NKRRK- 543
                                          * :::   .:*

Smittium culisis               ------------------------------------------------------------ 662
Pneumocystis murina            SLNEFSTENKQYITNTSTSLEFITQIPKGSEVSKEILDSSVNQSYSFNDKLVSKELYPIQ 519
Schizosaccharomyces pombe      --A-YTSQQLNP-APTAMPHP------------------NITSPSPSI----SVTQRPAV 578
Schizosaccharomyces octosporus --ASFTSTVNQN-ANTATSSS------------------SIQPNVPFFH--MSTPPPSTM 580

Smittium culisis               ------------------------------------------------------------ 662
Pneumocystis murina            VPPPPPYYSKPLTSSTENNLSLNSIE-----------------KKELQFLSSK------- 555
Schizosaccharomyces pombe      NVGPPPYVRPSAPSKLPD--TRQSIGDPLPPGAMANVSAGPSSVRSSSYNSTASESKSEI 636
Schizosaccharomyces octosporus TSVPPPYVRPPAHSQPRDSPYGRTIEDPLPPGAVAASSATSKSNTTL------------P 628

Smittium culisis               ------------------------------------------------------------ 662
Pneumocystis murina            ----------------------------------------------SYS----------- 558
Schizosaccharomyces pombe      TSHQNLHTIPINKPFTSDRPLYSSPNDTLERVETGNQGQRMNSIGNASSFSKRDIMENEN 696
Schizosaccharomyces octosporus GSSGQPGIVPNTSVLTSDRPLYVASHEANGERRPS------VSMSSYTAYSPSNGIQDKS 682

Smittium culisis               -------------------------------------------------   662
Pneumocystis murina            ------INSEST--------------------HKQGLKRPHEG------   575
Schizosaccharomyces pombe      --GSFDTNAKNGNNVDDSSSVRGMNLPSNSSDALRGVKRPLDETSSSYT   743
Schizosaccharomyces octosporus LHTTPSLHSERGGETNEGRPENHQDPMQSTGDVLRGVKRQFDEAPSSYT   731
```

Figure S1: Clustal W. Fhl1 polypeptides from different species were aligned using the Clustal W program (https://www.ebi.ac.uk/Tools/msa/clustalw2/), Fhl1 polypeptides from *Schizosaccharomyces pombe* (NP_594272.10), *Schizosaccharomyces octosporus* (XP_013017123.10), *Pneumocystis murina* (XP_007873813.10), and *Smittium culisis* (OMJ27350.160) were aligned by using the Clustal W program. The analysis shows that those polypeptides share two highly conserved regions corresponding to the FHA (light

blue) and FH (orange) domains. Symbols identification: "." semi-conserved amino acid substitutions in the column; ":" conserved amino acid substitutions in the column; "*" amino acid residues in that column are identical.
